# Osteopenia and Osteoporosis in Patients with Bronchiectasis: Association with Respiratory Parameters, Body Composition, Muscle Strength and Bone Remodeling Biomarkers

**DOI:** 10.1038/s41598-019-51069-0

**Published:** 2019-10-10

**Authors:** V. Contreras-Bolívar, G. Olveira, N. Porras, E. Acosta, E. Rubio-Martín, M. J. Tapia-Guerrero, J. Abuin-Fernández, C. Olveira

**Affiliations:** 1 UGC Endocrinología y Nutrición. Hospital Regional Universitario de Málaga/Universidad de Málaga. Instituto de Investigación Biomédica de Málaga (IBIMA), Málaga, Spain; 2 Centro de Investigación Biomédica en Red- Diabetes y Enfermedades Metabólicas asociadas (CIBERDEM), Barcelona, Spain; 3 UGC Neumología. Hospital Regional Universitario de Málaga. Hospital Regional Universitario de Málaga/Universidad de Málaga. Instituto de Investigación Biomédica de Málaga (IBIMA), Málaga, Spain

**Keywords:** Musculoskeletal system, Bone

## Abstract

The prevalence of osteopenia/osteoporosis has not been sufficiently studied in people with bronchiectasis not due to cystic fibrosis (BC), nor has its relationship with other variables (clinical, body composition and bone turnover and inflammation markers) been sufficiently studied. Our aim was to determine the prevalence of osteopenia and osteoporosis and related factors in patients with BC. We did a cross-sectional study in people with BC in a clinically stable state. Spirometric parameters, annual exacerbations and analysis with bone turnover markers (BTM) and inflammation markers were evaluated. Densitometry (DXA) was performed for body composition, bone mineral density (BMD) and handgrip strength. 123 patients were studied (65% women, mean age 49.6 ± 18.8, Body Mass Index (BMI) 24.8 ± 4.7 kg/m^2^). 62.8% and 62.5% of men and women, respectively, had normal bone mineral density, 30.2% and 22.2% osteopenia and 7% and 15% osteoporosis. 52 patients (56.2%) had low fat-free mass: 68.9% women and 28.6% men. Patients with decreased bone mass had significantly lower muscle strength, maximum expiratory volume in the first second (FEV1%), vitamin D, higher levels of C-terminal telopeptide of type 1 collagen (CTX) and total osteocalcin and underarboxylated osteocalcin (ucOC). We observed significant and negative correlations between BMD and the number of serious exacerbations per year CTX and undercarboxylated osteocalcin. We observed significant positive correlations between BMD, fat free mass index (FFMI) and handgrip dynamometry. The study suggest that the prevalence of osteoporosis was high in relation to the demographic characteristics. Respiratory parameters, body composition, muscle strength and bone remodeling markers were associated with a lower bone mineral density.

## Introduction

Bronchiectasis (BC) is not a disease in itself^1^, rather it is the end result of different diseases that have points of handling in common. They are due to the alteration of the ciliary epithelium and chronic inflammation of the bronchial wall causing abnormal and irreversible dilations of the cartilaginous bronchus, which are accompanied by the destruction of the muscular and elastic components of the bronchial wall^2^. BC is more frequent in women and its prevalence increases with age^3,4^. In the general population, patients with BC have a variable prevalence of between 25–272 cases per 100,000 inhabitants^5^. In Spain, a prevalence of 36.2 cases per 10,000 has been estimated, with an incidence of 4.81 cases per 10,000 inhabitants, predominantly in women^6^. The frequency of the different causes, as well as the percentage of BC considered idiopathic (24–53%) varies according to the series depending on age and geographical origin. In a recently published work in the Spanish population, it was demonstrated that the most frequent cause of BC is post-infection followed by idiopathic, cystic fibrosis (CF), primary immunodeficiencies and COPD (chronic obstructive pulmonary disease)^5^.

Vitamin D plays a key role in calcium homeostasis and bone metabolism^7^. An adequate intake of vitamin D and calcium contribute to bone health and reduce the risk of suffering osteopenia and osteoporosis; they are also the basis for the treatment of these disorders^8,9^. However, there is a high prevalence of deficit in calcium and vitamin D intake both in the general population and in patients with BC^10,11^.

BC patients have an increase in local and systemic inflammation. This systemic inflammation could be implicated in the incidence of osteopenia and osteoporosis^12^. In patients with BC, CF, and COPD, high levels of proinflammatory cytokines have been described, associated with an increase in muscle proteolysis, a decrease in lean mass and a greater number of exacerbations, even in stable patients^13–15^. In patients with COPD, systemic inflammation seems to be an independent predictor of low BMD^16^. The NHANES III study showed that the pattern of respiratory obstruction correlated independently with the decrease in bone mass^12^.

On the other hand, in patients with BC the prolonged taking of antibiotics is frequent, and there may be a sub-clinical deficit of vitamin K that is not usually evaluated^17,18^. In addition to coagulation, vitamin K acts as a co-factor for some proteins that are involved in bone mineralization and that could be used as markers of bone turnover such as osteocalcin and undercarboxylated osteocalcin^19–21^. Other markers have been proposed to assess bone resorption (such as C-terminal telopeptide of type 1 collagen –CTX- or receptor activator of nuclear factor kappa B ligand -RANKL-)^22,23^ meaning that the measurement of all these biomarkers could also be of interest to people with BC.

Both the possible deficits of vitamins D and K and the pro-inflammatory state and the decrease in fat-free mass and muscle strength which is observed in BC could condition a higher incidence and prevalence of osteoporosis and osteopenia in BC; however, there are very few studies which evaluate it^24^ and none that have assessed bone status in relation to body composition, muscle strength, lung function, bone turnover and inflammation biomarkers. Therefore, the aim of this study was to determine the prevalence of osteopenia and osteoporosis in a large sample of patients with BC and to evaluate the associated factors.

## Material and Methods

**Figure 1:**
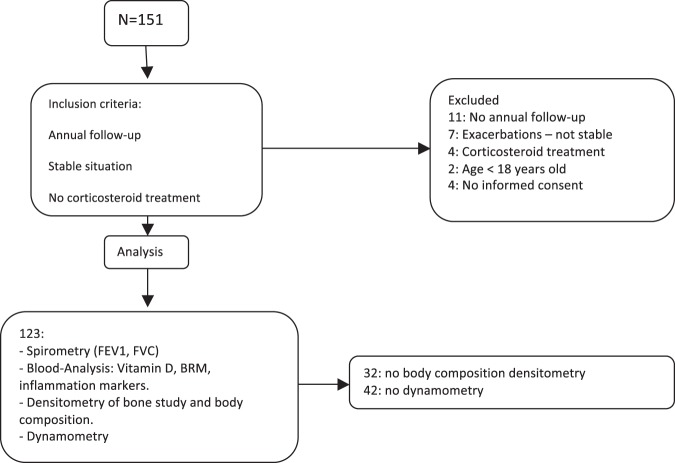
Flow chart. FEV1: maximum expiratory volume in the first second; FVC: functional residual capacity; BRM: bone remodeling markers.

The aim of the study was to determine the prevalence of osteopenia and osteoporosis and related factors in patients with BC. This is a cross-sectional observational study with prospective recruitment conducted between January 2016 and January 2017. A total of 123 patients were evaluated in the BC Unit of the Hospital Regional Universitario de Málaga (Fig. 1). The inclusion criteria were: to undergo regular follow-ups in the unit for at least two years and attend the annual study, to be aged 18 or over, to be in a situation of clinical stability, have not received oral corticoid treatment and having signed the informed consent. In all cases, BC patients were diagnosed by high resolution computerized tomography following the *Naidich et al*.^25^ criteria.

### Evaluation of bronchiectasis and respiratory situation

Forced spirometry *(Jaeger Oxycon Pro® pneumatograph*, *Erich Jaeger Würzberg*, *Germany)* was performed, determining values of functional residual capacity (FVC), maximum expiratory volume in the first second (FEV1) and the percentage ratio between both (FEV1/FVC)^26^.

Bronchorrhea, the amount of sputum produced per day expressed in millilitres, was evaluated according to the estimate of the patient during the three days prior to the visit^27^.

In the follow-up of all patients, the number of respiratory exacerbations is recorded prospectively. At the time of the annual study, respiratory exacerbations were recorded in the year prior to the evaluation. They were divided into mild-moderate (with oral antibiotic administration) and severe (when the administration of antibiotic treatment via intravenous route at home or with hospital admission was necessary).

### Dietary questionnaire

A 4 day dietary questionnaire of prospective dietary records was conducted (including one day of the weekend) according to the protocol published by our group to quantify calcium and vitamin D intake^28^. These data were analyzed with a computer application designed by our group for this purpose (*Dietstat*), and the food composition tables of *Jiménez and Mataix* were used^29,30^.

### Assessment of nutritional status and bone mineral density. Definition of osteoporosis

Dual energy densitometry (DXA) was performed using a Lunar Prodigy Advance densitometer (*General Electric Medical Systems*), providing information on total body composition (fat free mass, fat mass, bone mineral quantity -BMQ-) and bone mineral density (BMD) at the level of the lumbar spine (L2-L4) expressed in g/cm2, T-score and Z-score were compared with reference values^31^ and the diagnosis of osteopenia/osteoporosis was established according to WHO criteria and National Osteoporosis Foundation (NOF) guidelines^8,32,33^

The software used was EnCore 12.3 (*iDXA and Prodigy Advance*). The prevalence of low lean mass was assessed using the fat free mass index (FFMI: kg of lean mass/height^2^) according to ESPEN (European Society of Parenteral and Enteral Nutrition) criteria (<15 women or <17 men kg/m^2^)^34^. Muscle strength was assessed using *Jamar handgrip dynamometry (Asimow Engineering Co*., *Los Angeles*, *CA*), expressing the data in absolute figures and defining low strength values below the population 10th percentile^13,34,35^.

### Laboratory measurements

Blood samples were collected after a 12 hour fast, coinciding with the day of performing the bone densitometry. The extractions were completed after a clinical examination to confirm that the patients were in a stable phase. Plasma and serum were separated into aliquots and stored until analysis at −80 °C in the Hospital-IBIMA biobank, which forms part of the biobank of the public health system of Andalusia (BSSPA) and of the Spanish National Biobank Platform (PT13/0010/0006). Vitamin D was analyzed by electrochemiluminescent immunoassay (*Modular E-170*, *Roche Diagnostics*). The serum levels of inflammation and bone turnover biomarkers were determined by enzyme immunoassay techniques: Inflammatory cytokines (tumoral necrosis factor -alpha -TNFα-, Interleukin 6 -IL-6-) were measured using enzyme-immunoassay (EIA) (R&D Systems Europe Ltd). TNFα and IL-6 had intra-assay CV of 6 and 2,8% and inter-assay CV of 6% and 4,9% respectively. Serum levels of bone metabolism markers were determined by inmunoenzymatic microELISA assay. Undercarboxylated osteocalcin (TAKARA Bio INC, Japan) (intra-assay CV = 5,1%; inter-assay CV = 8,3%); Osteocalcin (R&D Systems Europe Ltd) (intra-assay CV = 3,1%; inter-assay CV = 7,2%); CTX (Serum CrossLaps, IDS-UK-)(intra-assay CV = 7,1%; inter-assay CV = 6,2%) and RANKL (InmmunoDiagnostics System,Germany) (intra-assay CV = 6.4%; inter-assay CV = 5.6%). All these determinations were quantified in Versamax (MTX Lab System, Barcelona, Spain).

### Data analysis

The Kolmogorov-Smirnov test was used to examine the normality of the quantitative variables that were expressed as the mean ± standard deviation. The differences between groups were analyzed by variance analysis adjusted for age and sex. Non-parametric tests (Mann-Whitney) were used when the variables to be analyzed did not follow a normal distribution. The qualitative variables were expressed as proportions, whose differences between groups were analyzed using the Chi-square test. The associations of variables were evaluated by estimating the Pearson or Spearman correlation coefficient (according to normality). For all calculations, a p probability of less than 0.05 significant for two tails was considered significant. The data analysis was performed with the SPSS 22.0 program (SPSS Inc., Chicago, IL, 2013).

### Ethics

The study was approved by the Provincial Research Ethics Committee of Málaga and all participants gave their written, informed consent.

### Ethics approval and consent to participate

The ethical principles contained in the latest revision of the Declaration of Helsinki and the standards of good clinical practice were applied. Confidentiality was guaranteed (Organic Law on Protection of Personal Data 15/1999), as well as the fact that all the information was only used for the purposes specified in the study. The study was approved by the Provincial Research Ethics Committee of Málaga and all participants gave their written, informed consent.

## Results

**Figure 2:**
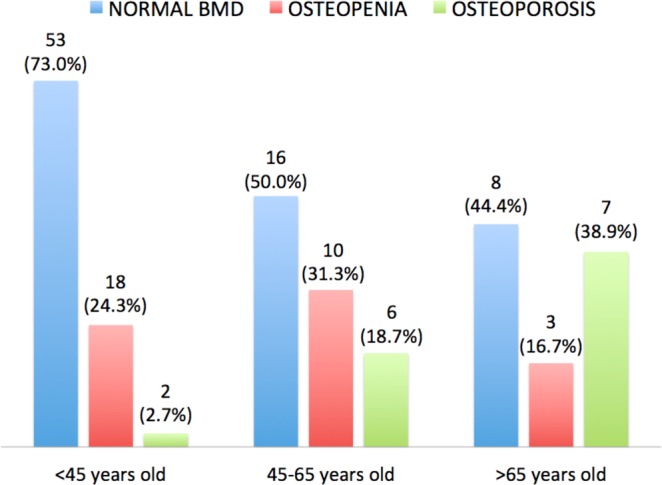
Osteopenia and osteoporosis proportion by groups of age. BMD: Bone Mineral Density.

123 patients were included who underwent bone densitometry in the lumbar spine. The general characteristics are shown in Table 1. 64.2% had normal BMD, 23.6% had osteopenia and 12.2% had osteoporosis. Only 3 patients who were women (2.4%) had suffered bone fractures due to osteoporosis (Table 2). We did not find significant differences between subjects with and without osteoporotic fractures. Patients diagnosed with osteoporosis and fractures vs. patients with osteoporosis without fractures presented: age 79.5 ± 6.8 vs 61.1 ± 16.4 (p = 0.84); FEV1 69.4 ± 27.6 vs 64.3 ± 22.2 (p = 0.78), exacerbations 2.3 ± 1.5 vs 2.4 ± 1.3 (p = 0.26); S.aureus colonization 0.43 ± 0.39 vs 1.0 ± 0.21 (p = 0.07), H.influenzae colonization 0.33 ± 0.49 vs 0.33 ± 0.57 (p = 1.0), colonization by P. aeruginosa 1.0 ± 0.11 vs 0.67 ± 0.49 (p = 0.28), T-score −3.1 ± 0.6 vs 3.4 ± 0.9 (p = 0.62). We found a prevalence of osteopenia of 23.6% and osteoporosis of 12.2%, increasing the latter progressively with age (2.3% in patients under 45, 18.7% in 45–65 and older than 65 years 38.9% (Fig. 2) and we observed a tendency (p = 0.08) in the subjects with fractures to have a higher age. 5 patients received previous treatment for osteoporosis with bisphosphonates, with a mean duration of 1.65 years (from 7 months to 2.6 years). Of the subjects who received treatment, 1 (20%) had osteopenia at the time of the densitometric study and 4 (80%) osteoporosis.

**Table 1:** General characteristics.

		BC (n 123)
Age	m ± SD	49.6 ± 18.8
<45 years	n (%)	54 (43.9)
45–65 years	n (%)	43 (35)
>65 years	n (%)	26 (21.1)
Age at diagnosis of BC	m ± SD	33.6 ± 18.3
Sex		
Men	n (%)	43 (35)
Women	n (%)	80 (65)
Smokers	n (%)	
No	n (%)	89 (72.4)
Yes	n (%)	12 (9.8)
Smoking index (pack-year)	m ± SD	16.7 ± 13.0
Ex-smokers	n (%)	22 (17.9)
Smoking index (pack-year)	m ± SD	24.8 ± 21.4
Etiology		
Post-infection	n (%)	44 (35.8)
Idiopathic*	n (%)	41 (33.3)
Cilliary dyskinesia	n (%)	25 (20.3)
Inmunodeficiency	n (%)	4 (3.3)
Collagen disease	n (%)	4 (3.3)
Other	n (%)	5 (4.0)
Treatment with PPI	n (%)	34 (40.5)
Treatment with ICS	n (%)	95 (77.2)
Respiratory status		
Daily broncorrhea (ml/day)	m ± SD	21.1 ± 27.2
S. aureus colonization	n (%)	34 (27.6)
H.influenzae colonization	n (%)	53 (43.1)
P.aeruginosa colonization	n (%)	70 (56.9)
Hypereactive airway disease	n (%)	109 (88.9)
Total exacerbations in the previous year	m ± SD	1.8 ± 1.4
	n (%)	98 (80)
Mild - moderate exacerbations	m ± SD	1.7 ± 1.4
	n (%)	95 (77.2)
Severe exacerbations	m ± SD	0.2 ± 0.5
	n (%)	13 (10.6)
FEV1 (%)	m ± SD	71.0 ± 23.0
>50%	n (%)	95 (77.2)
≤50%	n (%)	28 (22.8)
FVC (%)	m ± SD	75.0 ± 19.1
Azithromycin chronically**	n (%)	40 (32.5)
		**BC (n 123)**
Body composition by DXA (n = 91)		
Fat free mass index- FFMI (kg /m^2^) -		
Men	m ± SD	18.2 ± 1.7
Women	m ± SD	14.7 ± 1.6
Low FFMI	n (%)	50 (56.2)
Men	n (%)	8 (28.6)
Women	n (%)	42 (68.9)
Jamar dynamometry (kg) (n = 81)		
Mean		
Men	m ± SD	37.9 ± 11.4
Women	m ± SD	20.6 ± 5.6
Dinamometry/kg of FFM	m ± SD	0.62 ± 0.17
Low muscle strength		
Percentile ≥ 10	n (%)	66 (81.5)
Percentile <10	n (%)	15 (18.5)

m ± SD (m ± SD): mean ± standard deviation or (%).

BC: Bronchiectasis PPI: Proton pump inhibitors. ICS: Inhaled corticoid-steroid; FEV1: Expiratory volume in the first second. FVC: Forced vital capacity DXA: Densitometry, FFMI: Lean mass index.

*CFTR is included in Idiopathic: 29 patients (23.7) had a single mutation of the gene with negative sweat test and without fulfilling other criteria of clinical fibrosis.

**Three times a week for more than one year.

**Table 2:** Bone state.

		BC (n = 123)
BMD (g/cm^2^)	m ± SD	1.116 ± 0.225
Men	m ± SD	1.226 ± 0.187
Women	m ± SD	1.060 ± 0.224
BMQ (g)	m ± SD	2413.7 ± 556.3
Men	m ± SD	2993.7 ± 468.0
Women	m ± SD	2147.5 ± 355.5
T-score	m ± SD	−0.691 ± 1.672
Men	m ± SD	−0.576 ± 1.601
Women	m ± SD	−0.990 ± 1.631
Z-score	m ± SD	−0, 076 ± 1.323
Men	m ± SD	−0.003 ± 1.606
Women	m ± SD	−0.113 ± 1.169
Normal BMD	n (%)	77 (64.2)
Men	n (%)	27 (62.8)
Women	n (%)	50 (62.5)
Osteopenia	n (%)	31 (23.6)
Men	n (%)	13 (30.2)
Women	n (%)	18 (22.2)
Osteoporosis	n (%)	15 (12.2)
Men	n (%)	3 (7)
Women	n (%)	12 (15)
Antiresortive treatment (BP)	n (%)	5 (4.1)
Normal BMD	n (%)	0 (0)
Osteopenia	n (%)	1 (20)
Osteoporosis	n (%)	4 (80)

BC: Bronchiectasis BMD: Bone mineral density. BMQ: BMQ: Bone mineral quantity. BP: Bisphosphonates.

We evaluated if there were differences in relation to the smoking habit, there were no significant differences depending on the ex-smoker and smoker condition in the evaluated parameters. We did not find differences relating to the presence of colonizations by pathogens. We observed a tendency between presenting colonization with S.aureus and osteoporosis (p = 0.076)”.

42.3% (52) were supplemented with calcium and 55.3% (68) with vitamin D. 52.2% (64) had vitamin D levels greater than 30 mcg/dl, 27.8%^34^ between 20–30 mcg/dl and 20%^25^ less than 20 mcg/dl. In addition, we analyzed the levels of calcium and phosphorus and no patient presented alterations in the levels of these (Table 3).

**Table 3:** Clinical and analytical characteristics according to bone state.

		Normal BMD	Osteopenia	Osteoporosis	p
		(n = 77)	(n = 31)	(n = 15)	
Age	(m ± SD)	46.1 ± 17.1	49.1 ± 18.6	69.5 ± 11.2	0.382 ^a^ <**0**.**001**^**b**^ **0**.**009**^**c**^
Sex					
Men	n (%)	27 (62.8)	13 (30.2)	3 (7)	0.070
Women	n (%)	50 (62.5)	18 (22.5)	12 (15)	
FEV1 (%)	(m ± SD)	74.5 ± 22.6	69.9 ± 22.5	62.1 ± 12.8	**0**.**026**^**a**^ **0**.**017**^**b**^ 0.148^**c**^
Total exacerbations	(m ± SD)	1.8 ± 1.4	1.7 ± 1.6	2.4 ± 1.3	0.732^a^ 0.203^b^ 0.446^c^
Mild exacerbations	(m ± SD)	1.8 ± 1.4	1.6 ± 1.5	2.1 ± 1.1	0.729^a^ 0.394^b^ 0.241^c^
Serious exacerbations	(m ± SD)	0.08 ± 0.3	0.09 ± 0.3	0.27 ± 0.5	0.681^a^ 0.129^b^ 0.103^c^
BMI (kg/m^2^)	(m ± SD)	25.0 ± 5.2	24.9 ± 4.0	23.9 ± 38	0.055^a^ 0.275^b^ 0.280^c^
Fat free mass index (kg/m^2^)	(m ± SD)	16.1 ± 2.3	15.7 ± 2.4	14.4 ± 1.7	0.436^a^ 0.069^b^ 0.365^c^
Men	n 34 (%)	18.0 ± 1.8	18.6 ± 1.5	16.8 ± 0	0.484
Women	n 57 (%)	15.0 ± 1.9	14.2 ± 0.9	14.1 ± 1.6	0.173
Malnutrition according to FFMI	n = 52	27 (50.9)	15 (60)	10 (76.9)	**0**.**552**^a^ **0**.**136**^b^ **0**.**347**^c^
Mean dynamometry (kg)	(m ± SD)	27.0 ± 10.3	28.9 ± 14.5	17.9 ± 3.8	0.391^a^ 0.166^b^ 0.270 ^c^
Adjusted dynamometry per kg lean mass	(m ± SD)	0.61 ± 0.15	0.68 ± 0.19	0.51 ± 0.11	0.113^a^ **0**.**049**^**b**^ 0.266^c^
PPI	n (%)	18 (23.4)	6 (19.3)	7 (46.6)	0.572^a^ 0.074^b^ **0**.**048**^**c**^
Inhaled CTC	n (%)	51 (66.2)	20 (64.5)	12 (80)	0.956^a^ 0.684^b^ 0.442^b^
Calcium (mg/dl)	(m ± SD)	9,2 ± 0.5	9.4 ± 0.5	9.3 ± 0.5	0.357^a^ 0.714^b^ 0.746^c^
Phosphate (mg/dl)	(m ± SD)	3.3 ± 0.5	3.4 ± 0.5	3.6 ± 0.6	0.409^a^ 0.057^b^ 0.282^c^
25-OH-Vitamin D3 (mcg/dl)	(m ± SD)	33.9 ± 19.2	46.4 ± 34.7	30.7 ± 16.7	0.062^a^ 0.399^b^ **0**.**049**^**c**^
Undercarboxylated osteocalcin (ng/ml)	(m ± SD)	4.6 ± 2.4	5.1 ± 2.5	5.3 ± 2.4	0.159^a^ **0**.**022**^**b**^ 0.072^c^
Total osteocalcin (ng/ml)	(m ± SD)	25.0 ± 21.9	34.0 ± 36.8	54.2 ± 45.6	0.122^a^ **0**.**001**^**b**^ **0**.**036**^**c**^
CTX (mcg/ml)	(m ± SD)	0.46 ± 0.40	0.54 ± 0.39	0.72 ± 0.76	0.184 ^a^ **0**.**001** ^**b**^ 0.057 ^**c**^
RANKL (pmol/L)	(m ± SD)	0.30 ± 037	0.24 ± 0.31	0.15 ± 0.20	0.556^a^ 0.119^b^ 0.297^c^
IL-6 (pg/ml)	(m ± SD)	4.4 ± 6.1	3.4 ± 3.9	6.6 ± 9.6	0.355^a^ 0.436^b^ 0.301^c^
TNF-alpha (pg/ml)	(m ± SD)	4.6 ± 3.6	6.1 ± 5.1	7.0 ± 4.5	0.371^a^ 0.327^b^ 0.409^c^

m ± SD: mean ± standard deviation. ^a^Comparison between normal BMD and osteopenia. ^b^Comparison between normal BMD and osteoporosis. ^c^Comparison between osteopenia and osteoporosis (model adjusted for age and sex). FEV1: Expiratory volume in the first second. BMI: Body mass index. PPI: Proton pump inhibitors. CTX: C-telopeptide of type 1 collagen. RANKL: RANK ligand. IL-6: Interleukin 6. TNF-alpha: Tumoral Necrosis Factor-alpha.

*Patients who were under antiresorptive treatment were excluded from the analysis of bone remodeling markers.

Of the 123 patients, DXA for body composition was carried out in 91 patients and handgrip dinamometry in 81. However, we did not observe differences in the variables exacerbations, bronchorrhea, FEV1, bone status (BMD, BMQ and T-score) and BTMs among patients in whom the techniques were performed or not.“ 52 patients (56.2%) had low fat-free mass: 68.9% women and 28.6% men, according to the ESPEN criteria.

Table 3 shows the variables evaluated according to the presence or absence of osteopenia or osteoporosis. We found significant differences when adjusting for age and sex for FEV1 (%), vitamin D levels, CTX, human osteocalcin and undercarboxylated osteocalcin. In addition, we observed a significantly higher percentage of patients taking proton pump inhibitors (PPIs) in the osteoporosis group when compared with subjects who had osteopenia. There was a non-significant tendency to have a greater number of exacerbations, IL-6 levels and lower FFMI and muscle strength in subjects with osteoporosis.

The patients who took azithromycin chronically had significantly higher ucOC levels when compared with those who did not (5.3 ± 2.2 vs 4.7 ± 2.6, p = 0.037) without differences for total osteocalcin. 77.2% received treatment with inhaled corticosteroids (CTCs). There were no differences to those who did not receive inhaled CTCs in relation to BMD or the other clinical variables analyzed; only a tendency close to the significance p = 0.055 was observed of having higher levels of total osteocalcin in those who took vs. those who did not.

### Correlations between respiratory, densitometric, body composition and analytical variables

Negative correlations were observed between age with: BMD g/cm^2^ (r = −0.199, p = 0.039) and BMQ (r = −0.210, p = 0.048).

Significant and negative correlations were observed between the number of severe exacerbations per year and the T-score (r = −0.272, p = 0.019); between CTX and BMD g/cm^2^ (r = −0.227, p = 0.019), between undercarboxylated osteocalcin and Z-score (r = −0.299, p = 0.002) and between total osteocalcin and FVC (r = −0.193, p = 0.040).

We observed positive correlations between severe exacerbations and CTX (r = 0.254, p = 0.016); between the FFMI and between the handgrip dynamometry and BMD g/cm^2^ (r = 0.463, p < 0.001). We also found positive and significant correlations between vitamin D levels with FFM (r = 0.334, p < 0.001) and with the adjusted dynamometer per kg of lean mass (r = 0.314, p = 0.049).

## Discussion

Our study is the first to evaluate the prevalence of osteopenia and osteoporosis in a large sample of patients with BC and its association with multiple respiratory parameters, nutritional (body composition, diet and muscle strength) and analytical (vitamin D, bone turnover and inflammatory biomarkers).

65% of our sample were women and the average age of our sample was 49.6 years. The average age of our population is younger than that published in the literature for BC in the Spanish population^36^. In relation to osteoporosis, if we compare the general characteristics of the cohort to international cohorts or the Spanish Historical cohort, patients in the present cohort are younger (65 vs 50 years), and are more often females (55% vs 65%).

We found a prevalence of osteopenia of 23.6% and osteoporosis of 12.2%, increasing the latter progressively with age (2.3% in patients under 45, 18.7% in 45–65 and older than 65 years 38.9%).

There are few studies that evaluate the prevalence of osteoporosis without fractures in the Spanish population following the densitometric criteria. In the study carried out by *Diaz Curiel et al*., the prevalence of osteopenia was estimated as 31.9% and of osteoporosis as 4.3% in the age group 40 and 49 in women^31^. In another study carried out by same investigator, the prevalence of osteoporosis in the lumbar spine in women is: 0.34% in the group aged 20–44 years; 4.31% in the group aged 45–49 years; up to 9.09% in the group aged 50–59 years; 24.29% in the 60–69 years, and 40.0% in the group aged 70–79 years. The prevalence of osteoporosis in the femoral neck in women is: 0.17% in the group aged 20–44 years, 0% in the 45–49 years, up to 1.3% in the 50–59 years, 5.71% in the 60–69 years and 24.24% in the group aged 70–79 years^37^. The prevalence of post-menopausal osteoporosis in the general population increases with age from 15% for ages between 50 and 59 years to a prevalence greater than 80% for women over 80 years of age^38^. In our study, the population with BC is younger (therefore with a lower prevalence of post-menopausal osteoporosis) and has male representation. Thus, the results obtained in our work suggest that there could be a higher prevalence of osteoporosis than expected based only on demographic risk factors^10^. The proportion of males with osteopenia was 10.6% and with osteoporosis 2.4% of the total sample and 30.2% and 7% respectively, if only males were considered, with a mean age of 49.9 years. These data could be higher than those observed in males in the Spanish general population, with osteoporosis existing in only 1.1% of subjects with an average age of 65 years^39^.

In another study performed in 113 subjects with BC, 70 of whom had undergone a bone study, it was observed that more than 85% of the patients had a decrease in BMD, with 27% osteoporosis and 59% osteopenia. These figures are much higher than those described in the general population and those found in our study, possibly motivated by the higher average age (72 years) and the greater proportion of women (90% of the sample)^24^. In this study, 50.4% received treatment with PPI and 39.8% with inhaled CTC and no significant correlations with the decrease in BMD (osteopenia or osteoporosis) were found. In our work, most subjects received inhaled CTC (77.2%), however as in *Diehl’s* work, we did not find differences according to BMD. The prevalence of PPI consumption was 40.5% and we found a higher proportion of subjects undergoing PPI treatment in subjects with osteoporosis compared to those who had osteopenia. Although a non-significant trend was observed, we did not find differences when comparing them with those who had normal BMD.

BC are characterized by an increase in the respiratory tract and systemic inflammation^40,41^. The increase in systemic inflammation, determined by markers such as C reactive protein (CRP), IL-6 or TNF-alpha, has been associated with a decrease in bone mass in subjects with COPD^16^ and with decrease in fat free mass and a greater number of exacerbations, even in stable patients^11,12^. In our study, we observed an increase in the levels of IL-6 and TNF-alpha in subjects with osteoporosis, although they did not reach significant differences when compared with normal ones. However, we did observe significant correlations between severe exacerbations in the last year (clinical marker of inflammation) and bone mass, as well as lower spirometric values in patients with osteoporosis and osteopenia.

There are few studies in which the nutritional status in BC is evaluated, beyond the description of the BMI^15,42^. In the present work, the average BMI was normal, however, to make a more adequate approach to the assessment of nutritional status, it is also necessary to know the body composition^15^. We observed a high prevalence of low fat-free mass according to the criteria established by ESPEN, much higher in women (68.9%) when compared with men (28.6%). The FFMI was higher in those who had a normal BMD compared to those who had osteopenia and osteoporosis although it did not reach statistical significance. On the other hand, we found significant positive correlations between FFMI and handgrip dynamometry with BMD and between vitamin D levels with fat free mass and dynamometry. In addition, we observed, significantly, that patients with osteopenia had greater muscle strength than those who had osteoporosis as determined by dynamometry and adjusted per kg of FFM. *Rikkonen et al*. also found associations between fat-free mass and muscle strength and BMD in women with post-menopausal osteoporosis, being independent factors for the prediction of osteoporosis^43^. These results may be of clinical importance as the increase in lean mass and muscle strength prevents falls and stimulates bone formation^44^.

Vitamin D plays a key role in the regulation of calcium and bone homeostasis, as well as the increase in pro-inflammatory cytokines^7^. Low levels of 25-OH-vitamin D are also associated with muscle weakness and increased risk of falls^24,45^. In our study, we found a deficit of vitamin D of 20%, if the limit of normality is considered to be 20 mcg/dl, and of 47.8% if the limit is considered to be 30 mcg/dl, data similar to those available in the general population despite a non-negligible percentage of subjects were receiving supplementation^11^. Associations have been observed between low levels of vitamin D and BMD in different populations, including patients with COPD^24,45^ We only found this association when comparing subjects with osteopenia and osteoporosis, possibly because a high percentage received supplementation.

People with BC who are being treated with antibiotics for a long time, especially those with the highest number of exacerbations, may have a subclinical vitamin K deficit (due to a lower intestinal production of vitamin K2, when the microbiota is altered)^17,18^. In addition to coagulation, vitamin K acts as a co-factor for some proteins involved in bone mineralization such as osteocalcin (bone formation marker) and in the regulation of bone matrix calcification^19,46^. Vitamin K also inhibits the production of prostaglandin E2 and interleukin 6 (IL-6), which are known bone resorption agents^47^. In our study we observed that subjects with osteoporosis had higher levels of osteocalcin than subjects with normal BMD and osteopenia, probably in relation to an increase in bone turnover. We also found higher levels of undercarboxylated osteocalcin in patients with osteoporosis when compared with those with normal BMD. Given that patients with osteoporosis had a greater number of respiratory exacerbations, that patients receiving regular treatment with Azithromycin had significantly higher levels of undercarboxylated osteocalcin, and considering that levels of undercarboxylated osteocalcin rise with vitamin K deficiency^48^, it could be speculated that the higher consumption of antibiotics could predispose a sub-clinical vitamin K deficiency and a greater loss of bone mass.

Bone turnover markers predict the rate of bone formation and destruction and in some studies have been linked to the risk of fracture^20^. Subjects with osteoporosis had significantly higher levels of CTX but not RANKL when compared with those with normal BMD and osteopenia. We also observed significant associations between CTX levels, BMD, and the number of serious exacerbations, which points to its possible usefulness in the diagnosis and follow-up of these patients.

The strengths of the study include the fact that it is the first to evaluate the prevalence of osteopenia and osteoporosis in a large sample of patients with BC, assessing its association with multiple parameters such as body composition (lean mass status), muscle strength, respiratory parameters, state of vitamins D and K and bone turnover and inflammation biomarkers. However, it is not without limitations: it is a single centre and cross sectional study, which prevents us from drawing causal conclusions, so we can only speculate with different associations. The measurement of vitamin D was not carried out homogeneously at the end of winter.

## Conclusion

In conclusion, the prevalence of osteoporosis was high in our serie. Respiratory parameters, fat-free mass, muscle strength and markers of bone turnover were associated with lower bone mineral density. The conducting of prospective studies will allow a more extensive consideration of these associations.

## Data Availability

The datasets generated and/or analyzed during the current study are not publicly available as they are being analyzed in another study, however they are available from the corresponding author on reasonable request.
